# Anti-Inflammatory Property of the Essential Oil from *Cinnamomum camphora* (Linn.) Presl Leaves and the Evaluation of Its Underlying Mechanism by Using Metabolomics Analysis

**DOI:** 10.3390/molecules25204796

**Published:** 2020-10-19

**Authors:** Jiali Chen, Cailin Tang, Yang Zhou, Rongfei Zhang, Shaoxia Ye, Zhimin Zhao, Ligen Lin, Depo Yang

**Affiliations:** 1 School of Pharmaceutical Sciences, Sun Yat-Sen University, Guangzhou 510006, China; chenjli26@mail2.sysu.edu.cn (J.C.); tangclin@mail2.sysu.edu.cn (C.T.); zhouy596@mail2.sysu.edu.cn (Y.Z.); zhangrf7@mail2.sysu.edu.cn (R.Z.); yeshx9@mail2.sysu.edu.cn (S.Y.); zhaozhm2@mail.sysu.edu.cn (Z.Z.); 2 State Key Laboratory of Quality Research in Chinese Medicine, Institute of Chinese Medical Sciences, University of Macau, Avenida da Universidade, Taipa, Macau 999078, China

**Keywords:** essential oil, *Cinnamomum camphora* (Linn.) Presl, anti-inflammation, BV2 microglial cells, metabolomics, glycolysis

## Abstract

*Cinnamomum camphora* (Linn.) Presl has been widely used in traditional Chinese medicine for a variety of purposes. Our previous study indicated the antibacterial mechanism of the essential oil (EO) from *C. camphora* leaves; however, its anti-inflammatory activity and the underlying mechanism have not been clearly demonstrated. Thus, the present study investigated its anti-inflammatory property. Our data revealed that EO significantly decreased the release of nitric oxide (NO) and the mRNA expression of inducible NO synthase (iNOS) in lipopolysaccharide (LPS)-induced BV2 microglial cells. EO also attenuated LPS-induced increase in the mRNA expression and secretion of inflammatory cytokines including interleukin-6 (IL-6), IL-18, IL-1β and tumor necrosis factor-α (TNF-α). Furthermore, the metabolic profiles of LPS-induced BV2 microglial cells treated with or without EO were explored. Thirty-nine metabolites were identified with significantly different contents, including 21 upregulated and 18 downregulated ones. Five pathways were enriched by shared differential metabolites. Compared with the control cells, the glucose level was decreased, while the lactate level was increased, in the culture supernatant from LPS-stimulated cells, which were reversed by EO treatment. Moreover, compared to the LPS-treated group, the activities of phosphofructokinase (PFK) and pyruvate kinase (PK) in EO group were decreased. In summary, the current study demonstrated that EO from *C. camphora* leaves acts as an anti-inflammatory agent, which might be mediated through attenuating the glycolysis capacity of microglial cells.

## 1. Introduction

*Cinnamomum camphora* (Linn.) Presl, commonly known as camphor tree or camphor laurel, is widely cultivated in Southern China as an ornamental plant and a material for wood furniture [1]. In traditional Chinese medicine, the essential oil from this plant (EO) has been widely used to treat inflammation, rheumatic conditions, and muscular strains [2]. A growing number of pharmacological researches have shown that *C. camphora* exhibits a wide range of biological activities such as anti-inflammation, anti-bacteria, antioxidation, anti-fungi, and repellent properties [3,4,5,6,7,8,9,10,11]. In our previous study [12], 48 constituents from EO were identified; linalool was the major compound, followed by eucalyptol, isoborneol, α-terpineol, and camphor (Supplementary Material, Table S1).

Inflammation is self-protective responses of body tissues to harmful stimuli, such as pathogens, damaged cells, or irritants [13]. Dysregulated inflammation, such as long-term chronic inflammation, has been considered to play key roles in the onset and development of metabolic disorders, neurodegeneration, and cardiovascular diseases [13,14]. Recently, many studies investigated that microglia-mediated neuroinflammation plays an essential role in the pathophysiological process of multiple neurological disorders [15,16,17]. It is of great significance to attenuate the increases of various inflammatory mediators, including tumor necrosis factor-α (TNF-α), interleukin (IL)-6, IL-1β, and nitric oxide (NO) [18,19,20]. In addition, inflammatory response is usually associated with metabolic changes [21]. Metabolomics is considered to be a powerful platform to explore the potential mechanisms of drugs at the biochemical level. A recent study investigated the anti-neuroinflammatory potential of *Clinacanthus nutans* leaf extracts towards lipopolysaccharide (LPS)-induced BV2 microglial cells by using metabolomics approach [22]. Moreover, effective therapeutic drugs for neuroinflammation are lacking. The anti-inflammatory activity of EO and its potential mechanisms remain largely unelucidated. Therefore, it is of significance to use BV2 microglia cells to study the anti-inflammatory effect of EO.

In recent years, increasing evidence indicates that glycolysis plays an important role in the development of inflammation [23,24]. Glycolysis, a ubiquitous metabolic pathway in mammals, is regulated by multiple glycolytic enzymes, including phosphofructokinase (PFK) and pyruvate kinase (PK) [25,26]. Activation of the glycolytic pathway provides essential biomacromolecules, including amino acids and fatty acids, to synthesize inflammatory mediators in cells. LPS-induced cells undergo changes in glycolytic metabolism [27]. The increased level of lactate is also related to glycolytic metabolism [28,29]. Furthermore, lactate has been reported to enhance LPS-stimulated proinflammatory responses [30]. Therefore, the anti-inflammatory activity of molecules might be related to their effect on glycolytic pathway. A previous study showed that lidocaine inhibits the secretion of inflammatory cytokines in LPS-induced peritoneal macrophages by inhibiting the glycolytic pathway [31].

In order to assess the anti-inflammatory activity of EO and explore the underlying mechanisms, this study was designed to detect NO production, cytokines secretion, and mRNA expression of inducible NO synthase (iNOS), IL-6, IL-18, IL-1β, and TNF-α in LPS-induced BV2 microglial cells. Furthermore, GC-MS-based metabolomics analysis and the glycolytic enzyme activity were investigated. Herein, we desire to provide better insight into the anti-inflammatory potential of EO.

## 2. Results

### 2.1. Cytotoxic Effect of EO on BV2 Microglial Cells

**Figure 1:**
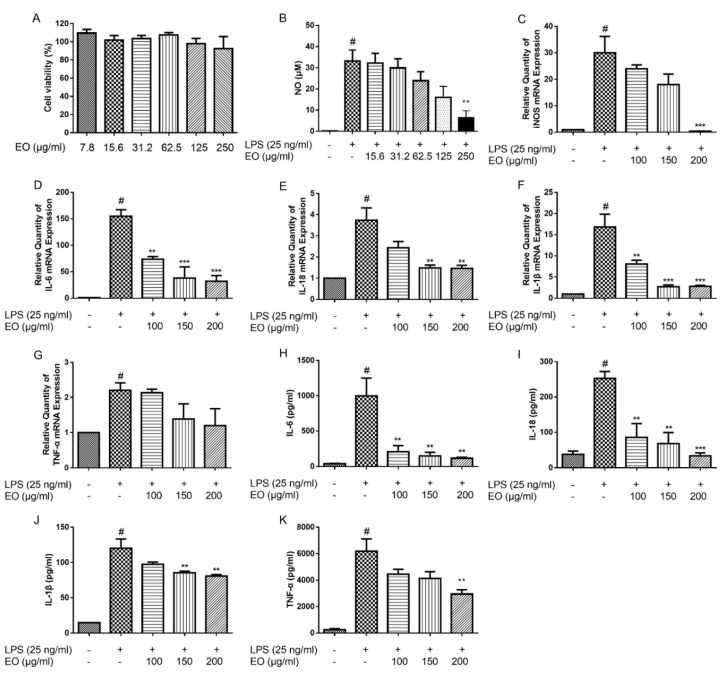
Effect of essential oil (EO) on the viability of BV2 microglial cells (**A**). Effects of EO on lipopolysaccharide (LPS)-induced production of nitric oxide (NO) (**B**) and mRNA expression of iNOS (**C**). Effects of EO on LPS-induced mRNA expression levels of IL-6 (**D**), IL-18 (**E**), IL-1β (**F**), and TNF-α (**G**) in BV2 microglial cells, and secretion of IL-6 (**H**), IL-18 (I), IL-1β (**J**), and TNF-α (**K**) in the culture supernatant from BV2 microglial cells. (^#^ *p* < 0.05 vs. control, ** *p* < 0.01, *** *p* < 0.001 vs. LPS treatment, *n* = 3).

To exclude the cytotoxicity, the BV2 microglial cells were treated with EO at concentrations ranging of 7.8‒250 µg/mL for 48 h. The viability of BV2 microglial cells was measured by the MTT assay. The results showed that EO did not obviously affect cell viability even at a concentration up to 250 µg/mL (Figure 1A). Thus, the concentrations of 7.8‒250 µg/mL of EO were selected for further experiments.

### 2.2. Effects of EO on the Production of NO and the Expression of iNOS

In order to investigate the anti-inflammatory effect of EO, NO production was evaluated in LPS-induced BV2 microglial cells. As shown in Figure 1B, the NO production in LPS exposed cells was significantly increased, compared with the control cells, whereas EO treatment concentration-dependently decreased NO production in BV2 microglial cells. Subsequently, RT-PCR results showed that EO suppressed the LPS-induced increase of iNOS expression in a concentration-dependent manner (Figure 1C). These results indicated that EO inhibited NO production through downregulating iNOS mRNA level in LPS-induced BV2 microglial cells.

### 2.3. Effects of EO on the Expression and Secretion of IL-6, IL-18, IL-1β, and TNF-α

To confirm the anti-inflammatory effect of EO, the levels of inflammatory mediators, including IL-6, IL-18, IL-1β, and TNF-α, were evaluated on LPS-stimulated BV2 microglial cells. Compared with the control group, LPS increased IL-6, IL-18, IL-1β, and TNF-α transcriptions, which were decreased after treated with EO in concentration-dependent manners (Figure 1D‒G). The ELISA results indicated that the secretion of IL-6, IL-18, IL-1β, and TNF-α in cell supernatants were remarkably increased in LPS group compared with the control; and EO treatment alleviated the elevated levels of IL-6, IL-18, IL-1β, and TNF-α (Figure 1H‒K).

### 2.4. Metabolomics Analysis

**Figure 2:**
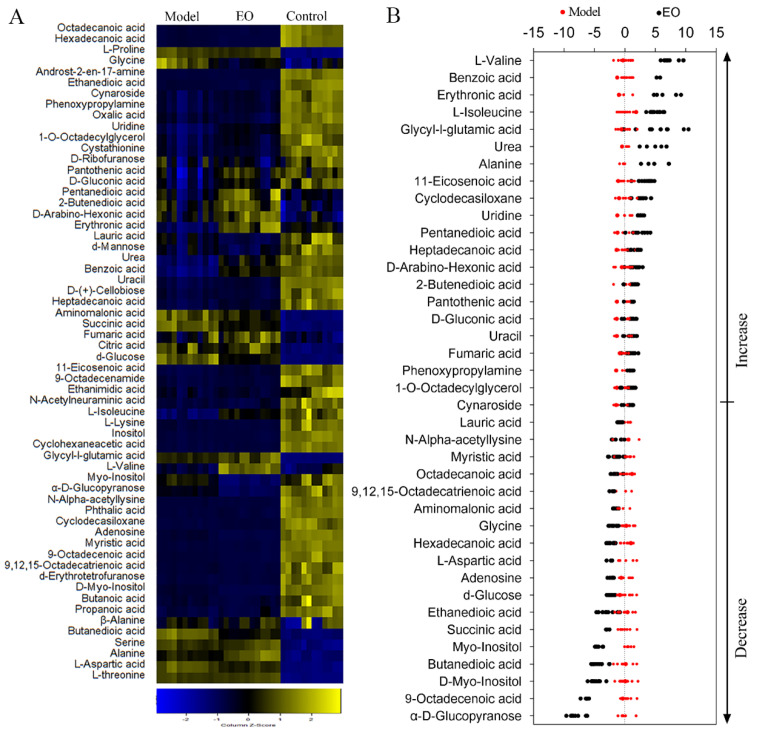
Metabolic profiles of BV2 microglial cells in response to EO. (**A**) Heat maps of metabolites (row). Yellow and blue indicate an increase and decrease of the metabolites relative to the mean and standard deviation of the row metabolite level, respectively (see color scale). (**B**) Z-score scatter diagrams of significant differentially expressed metabolites (*p* < 0.05) in the presence or absence of EO. Metabolites are showed on the y-axis.

Six biological and two technical replicates yielded 36 data points in this study. There were 60 metabolites for subsequent analysis after data processing. KEGG (Kyoto Encyclopedia of Genes and Genomes) was used to retrieve the biological categories of the identified metabolites. The cells treated with LPS alone were set as model group, and those treated with neither EO nor LPS were set as control group. The abundance of 39 metabolites in the EO group was different in comparison of the model group (Figure 2A). The Z-value was calculated for a comparative study based on the model group, showing the variations of these metabolites. The Z-score plot displayed that it spanned from −7.74 to 7.37 in the EO group (Figure 2B). Specifically, there were 18 metabolites decreased and 21 metabolites increased in the EO group, when compared with the model group.

**Figure 3:**
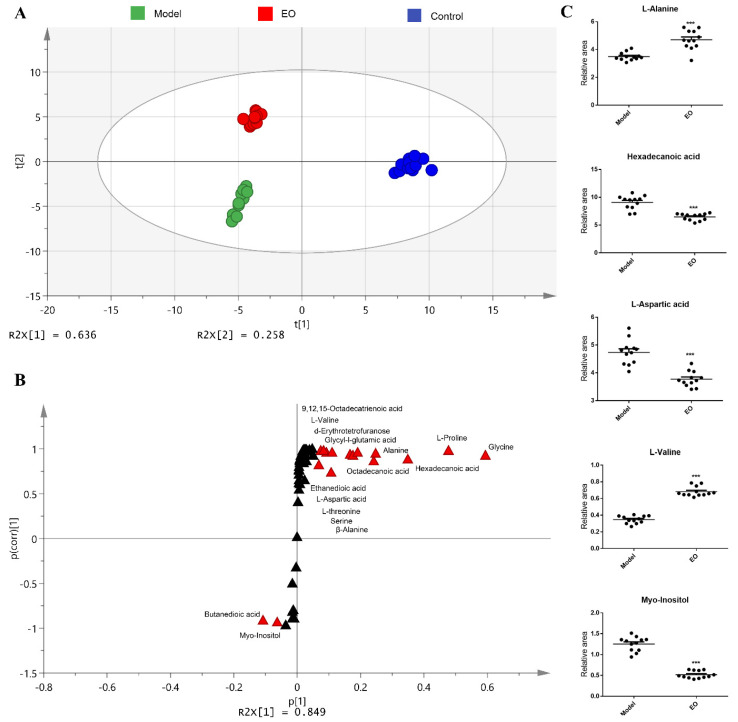
(**A**) Principal Component Analysis (PCA) analysis of three groups. Each dot represents the technological replicate analysis of samples. (**B**) S-plot generated from orthogonal partial least-squares discriminant analysis (OPLS-DA). Dot represents metabolites, which are *p* [1] < −0.05 or > 0.05 and *p* (corr) [1] < −0.5 or > 0.5 and marked in red dot. (**C**) Scatter diagram of five biomarkers, each dot shows a technical replicate. *n* = 6. *** *p* < 0.001 vs. model group.

To investigate the metabolic differences among the control group, model group, and EO group, orthogonal partial least-squares discriminant analysis (OPLS-DA) was applied for the recognition of the sample patterns, followed by ranking the altered metabolites in loading. The three groups were separated obviously (Figure 3A). Discriminating variables were present in the S-plot (Figure 3B), while cut-off values were set as greater or equal to the 0.05 and 0.5 for the absolute value of covariance p and correlation p (corr), respectively. Alanine, hexadecanoic acid, aspartic acid, valine, and myo-inositol showed the largest correlations and covariances in the predictive component between the model group and the EO group (Figure 3C).

**Figure 4:**
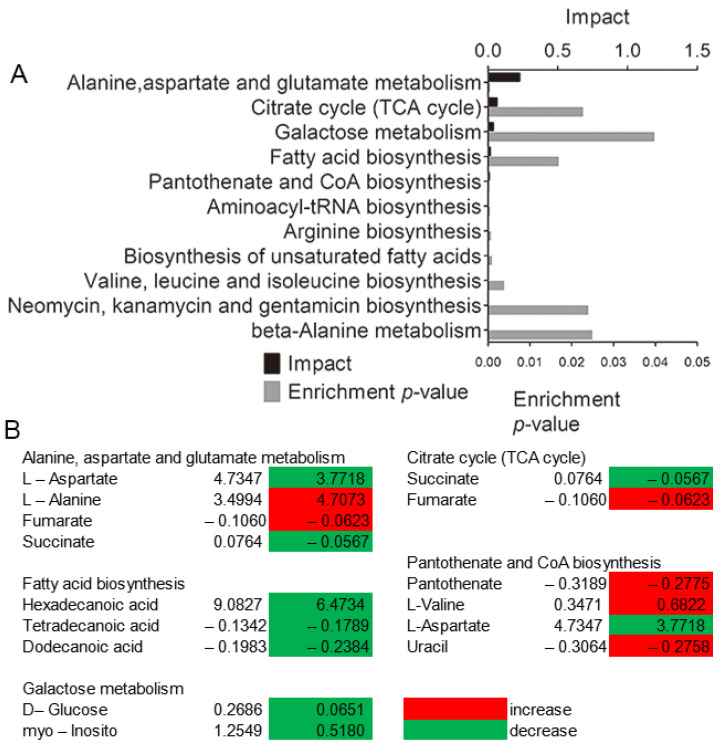
(**A**) Enriched pathways in the presence of the EO significantly enriched pathways were plotted. (**B**) Heat map of the average levels of differential metabolites in the five enriched pathways shared by the two groups. Red and green, respectively, indicate increase and decrease in the metabolites scaled to mean and standard deviation of row metabolite level (see the color scale).

Five impacted pathways were enriched by shared differential metabolites, including alanine, aspartate and glutamate metabolism, tricarboxylic acid cycle (TCA cycle), galactose metabolism, fatty acid biosynthesis, and pantothenate and Coenzyme A (CoA) biosynthesis, between the model and EO groups (Figure 4A). The first four pathways were dramatically impacted (*p* < 0.05), where amino acid metabolism was the most significant one, followed by glucose metabolism. As shown in Figure 4B, most of metabolites enriched in the five pathways were decreased. The alanine aspartate and glutamate metabolism pathway was mostly impacted among the altered metabolic pathways under EO treatment (*p* < 0.05). These results indicated that anti-inflammatory activity of EO might be through interfering with amino acid and glucose metabolism.

### 2.5. Analysis of Lactate and Glucose Levels

**Figure 5:**
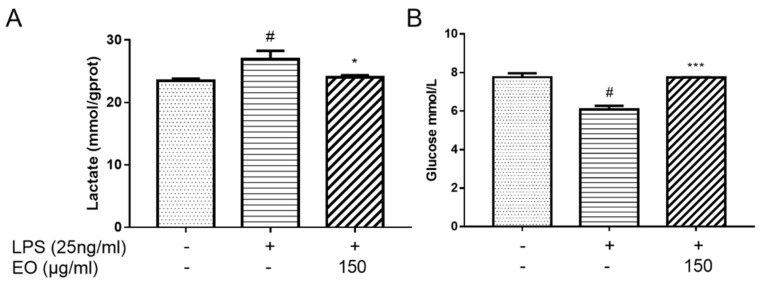
Measurement of the lactate (**A**) and glucose (**B**) levels in the supernatant of BV2 microglial cells. ^#^ *p* < 0.01 vs. control; * *p* < 0.05; *** *p* < 0.001 vs. model group. All tests were performed in triplicate.

To evaluate whether glycolytic activity was suppressed by EO treatment, lactate production, and glucose uptake were detected in BV2 microglial cells. Compared with the control group, the glucose level was decreased, while the lactate level was increased, in the culture supernatant of LPS-induced BV2 microglia cells, indicating that LPS treatment augmented the glycolytic capacity; however, the glucose level was increased and the lactate level was decreased after EO treatment, which indicated that EO suppressed the glycolytic capacity induced by LPS (Figure 5).

### 2.6. Enzyme Activity Analysis

**Figure 6:**
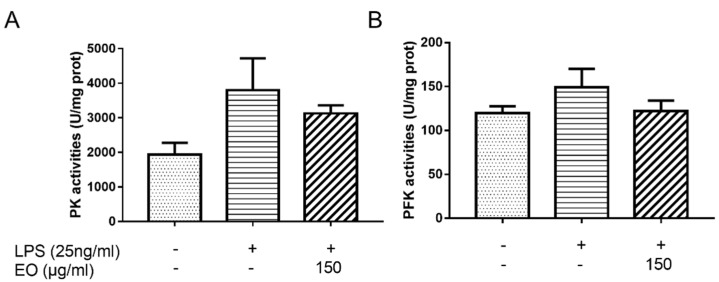
Measurement of the EO on PK (**A**) and PFK (**B**) activities in BV2 microglial cells. All tests were performed in triplicate. All tests were performed in triplicate.

To further demonstrate the effect of EO on glycolysis, the activities of PK and PFK, key enzymes associated with glycolysis, were measured. As shown in Figure 6, the PK activities for the control, model, and EO groups were 1940.37, 3798.00, and 3129.82 U/mg prot, respectively; the PFK activities for the control, model, and EO groups were 119.57, 149.32, and 122.10 U/mg prot, respectively. Compared to the control group, the activities for PK and PFK increased by 95.74% and 24.88% after treatment with LPS, respectively, whereas the activities for PK and PFK decreased by 17.59% and 18.23% after EO treatment. It suggested that EO could disturb the LPS-induced activation of glycolysis.

## 3. Discussion

Inflammation, an important protective response of organism, aims to eliminate or scavenge harmful stimuli, including damaged cells and pathogens, so as to restore normal tissue structure and function. A wild inflammation causes harmful effects to tissues and microcirculatory systems, and leads to severe complications [13]. Therefore, alleviation of inflammatory responses is the key for tissues to recover from different stimulating factors [32]. NO plays an important role in the immune system and the maintenance of body quiescence, but excessive NO causes a variety of inflammatory related diseases. In addition, the upregulation of inflammatory factors persisting at an inflammatory site is important, such as IL-6, IL-18, IL-1β, and TNF-α. Overproduction of these mediators recruits downstream effector cells (such as neutrophils), and directs the natural evolution of the inflammatory response [33]. Therefore, it is of great significance to explore the mechanisms of inflammation by detecting and observing the changes of these inflammatory factors [34]. The model of LPS-induced NO production in BV2 microglial cells is widely employed in screening anti-inflammatory agents for neuroinflammation and related neurodegenerative diseases [35]. Kim et al. found that ginsenosides exert anti-inflammatory activities by suppressing the inflammatory enzymes, such as iNOS and COX-2, as well as the production of pro-inflammatory cytokines such as TNF-α, IL-1β, and IL-6, in LPS-stimulated macrophages and microglial cells [36]. Currently, EO was found to inhibit LPS-induced NO production via suppression of iNOS expression at the mRNA level. In addition, the production of pro-inflammatory cytokines (IL-6, IL-18, IL-1β, and TNF-α) was significantly reduced by EO in BV2 microglial cells. These results showed that EO notably inhibits the expression of pro-inflammatory mediators, suggesting that it could inhibit the occurrence of inflammatory responses or reduce the aggravation of inflammatory mediators.

Recent findings suggested that inflammation is closely related to metabolic disorders [37,38,39]; thus, the metabolites of biological organisms can be used as important indicators to reflect the inhibition or activation of related metabolic pathways under inflammation. The significantly different metabolites can be witnessed by metabolomics profiling analysis [24]. Previous investigations have shown that LPS activates BV2 microglial cells resulting in altered metabolism. Therefore, this study applied metabolomics to elucidate the anti-inflammatory mechanism of EO in LPS-induced BV2 microglial cells. A previous study showed that the changes in the metabolite levels were related to the anti-inflammatory effects of volatile oils from *Angelica sinensis* (VOAS), after the intervention of VOAS in acute inflammation displayed a trend of restoring the level of biomarkers to normal [40]. Similarly, the relative concentrations of metabolites between the model group and the EO group further demonstrated that physiological metabolism of LPS-induced BV2 microglial cells was restored after EO treatment. According to the metabolomics analysis, most were amino acids among the 39 significantly different metabolites, which play essential roles in an organism. Further enrichment analysis and pathway analysis pointed mainly to the involvement of amino acid, glucose metabolism, and fatty acid metabolism. Based on metabolic pathway analysis, the TCA cycle plays the most important role in the amino acid metabolism, and the major metabolic part of galactose metabolism is glycolysis [40].

Recent studies have shown that pro-inflammatory responses of microglia are driven by glycolysis, which is associated with high level of glucose uptake, revealing the crucial function of glycolysis in inflammation responses [19]. Lactate is an important product of glycolysis, and a high lactate level is associated with a number of cellular events that lead to inflammation [30]. Therefore, the contents of glucose and lactate in the cell supernatant can reflect the glycolytic capacity of BV2 microglial cells. The experimental results showed that EO inhibits LPS-induced increase of glucose consumption and lactate production, suggesting that EO might inhibit the glycolytic ability in LPS-induced BV2 microglia cells. Growing evidence indicates that glycolysis is closely linked with several key enzymes, which are crucial for the regulation of glycolysis, including PFK and PK [21]. Wang et al. demonstrated that the deacetylase Sirtuin5 plays a role in regulating inflammatory response by regulating the PK activity of PKM2; moreover, withaferin A, a bioactive compound derived from *Withania somnifera*, considerably improved hepatic inflammation by regulating glycolysis-related enzyme genes in the liver of obese mice [41]. Similarly, our results verified that cells treated with EO suffered a decrease in the activities of PK and PFK and disrupted glycolysis. Glycolysis converts glucose into pyruvate, which is subsequently converted into lactate, and secreted, or gets into the TCA cycle, which pointed out the importance of glycolysis. Therefore, further study about the factors affecting glycolysis can be the direction of our research.

## 4. Materials and Methods

### 4.1. Reagents

3-(4,5-dimethylthiazol-2-yl)-2,5-diphenyltetrazolium bromide (MTT), dimethyl sulfoxide (DMSO), trypsin, and LPS were acquired from Sigma-Aldrich (St. Louis, MO, USA). Penicillin and streptomycin were purchased from Hyclone (Logan, UT, USA). Dulbecco’s modified Eagle medium (DMEM), fetal bovine serum (FBS), and Trizol RNA extraction reagent were obtained from Invitrogen (Grand Island, NY, USA). The BCA protein kit and NO assay kit were obtained from Beyotime Institute of Biotechnology (Shanghai, China). The ELISA kits for IL-18, IL-1β, and IL-6 were purchased from Boster Biological Technology (Wuhan, China). Hieff™ qPCR SYBR^®^ Green Master Mix (Yisheng, Shanghai, China), glucose and lactate assay kits were acquired from Nanjing Jiancheng Bioengineering Institute (Nanjing, China). The PFK and PK activity assay kits were purchased from Beijing Solarbio Science & Technology Co., Ltd. (Beijing, China).

### 4.2. Sample Collection and Extraction

Leaves of *C. camphora* were collected from adult trees in the campus of Sun Yat-Sen University, on March 2019, and identified by Professor Depo Yang, School of Pharmaceutical Sciences, Sun Yat-Sen University. A voucher specimen for *C. camphora* (NO. A20190301) has been deposited at the School of Pharmaceutical Sciences, Sun Yat-Sen University. The EO was extracted by hydrodistillation in a Clevenger-type apparatus for 2 h, and then dehydrated by filtration with anhydrous sodium sulfate.

### 4.3. Cell Culture

The BV2 microglial cells were obtained from the Cell Bank of Shanghai Institute of Biochemistry and Cell Biology (Chinese Academy of Sciences, Shanghai, China). Cells were cultured in DMEM with 10% FBS, 100 U/mL penicillin, and 100 g/mL streptomycin in a humidified incubator (Thermo Fisher, San Diego, CA, USA) containing 5% CO_2_ at 37 °C. A TPVG solution (0.2% trypsin, 0.05% glucose and 0.2% EDTA in phosphate buffer saline solution) was used for cell dissociation.

### 4.4. Determination of Cell Viability

BV2 microglial cells (5.0 × 10^3^ cells/well) were incubated in a 96-well plate for 24 h and then treated with EO at different concentrations. After 48 h, cell viability was measured by MTT assay as described in previously reported protocol with a little bit adjustment [42,43]. In brief, 20 µL of 5 mg/mL MTT was added to each well and the cells were incubated at 37 °C in the dark. After 4 h, the supernatant was removed. Then, the formazan crystals were dissolved in 100 µL of DMSO, after which the absorbance was measured at 490 nm.

### 4.5. Measurement of NO Production

BV2 microglial cells (5.5 × 10^4^ cells/well) were incubated in a 96-well plate for 24 h and then treated with EO at different concentrations in the absence or presence of LPS (25 ng/mL). The cells treated with LPS alone were set as the model group, and those treated with neither EO nor LPS were set as the control group. After 24 h, NO production was determined by measuring the amount of nitrite in cell culture supernatants using the nitric oxide assay kit.

### 4.6. Measurement of IL-6, IL-18, IL-1β and TNF-α Secretion

BV2 microglial cells (5 × 10^4^ cells/well) were seeded in a 12-well plate and incubated for 24 h. After treated with EO at different concentrations (100, 150, 200 µg/mL) and stimulated with LPS (25 ng/mL) for 24 h, the release of IL-6, IL-18, IL-1β, and TNF-α in the culture supernatants was quantified by using the corresponding ELISA kits according to the manufacturers’ protocols.

### 4.7. Total RNA Extraction and RT-PCR

BV2 microglial cells (1 × 10^6^ cells/well) were incubated into 6-well plates for 24 h. The cells were treated with different concentrations of EO in the presence or absence of LPS (25 ng/mL) for 24 h. The RNA samples were isolated with Trizol reagent, and reversely transcribed into complementary DNA using HiScript II Q RT SuperMix for qPCR. Quantitative RT-PCR was performed using the Hieff™ qPCR SYBR^®^ Green Master Mix according to the manufacturer’s protocol and detected by the LightCycler 96 Real-Time PCR System. All experiments were performed in triplicate. Relative mRNA levels were calculated using the 2^−ΔΔCt^ method. GAPDH was used as a housekeeping gene. The primer sequences are shown in Table 1.

### 4.8. Metabolomics Analysis

BV2 microglial cells were collected by centrifugation at 1000 rpm for 3 min, washed with PBS three times, and resuspended in methanol. The cells were homogenized by ultrasonic, followed by centrifugation at 12,000 rpm at 4 °C for 10 min. The supernatant of each sample was collected, and 10 μL ribitol (0.1 mg/mL) was added as the internal standard for analysis. Then, the supernatants were concentrated in a rotary vacuum centrifuge device (LABCONCO). The dried extracts were used for GC-MS analysis, as described in a previous study [12]. In brief, samples were derivatized. A 90 min reaction at 37 °C with 80 μL of 20 mg/mL methoxyamine hydrochloride in pyridine was carried out to protect carbonyl moieties. Then, derivatization of acidic protons was performed at 37 °C for 30 min with the addition of 80 μL of *N*-methyl-*N*-(trimethylsilyl) trifluoroacetamide (MSTFA). The derivatized sample (1 μL) was injected into a DB-5MS capillary column (30 m × 250 µm × 0.25 µm) by using the splitless model, and the analysis was fulfilled on an Agilent 7890A/5975MSD system. The temperature of the GC oven was first maintained at 70 °C for 5 min, then increased to 270 °C at a rate of 2 °C/min, and finally kept at 270 °C for 5 min. Helium was used as the carrier gas with a flow rate of 1 mL/min. The electron impact ionization was of 70 eV energy. The mass spectrometer was worked at 60–600 *m/z*. Each sample contained six biological and two technical replicates.

### 4.9. Measurement of Lactate and Glucose Levels

BV2 microglial cells were treated under the same conditions as those of RNA extraction. Lactate and glucose levels in the culture supernatants were determined by using the lactate and glucose assay kits, respectively.

### 4.10. Measurement of Enzyme Activity

Cells were collected by centrifugation at 1000 rpm for 3 min and then washed with PBS three times. The PFK and PK activities were analyzed spectrophotometrically by using commercially available kits according to manufacturer’s instructions. Inhibition rate % = [(sample − control)/control] × 100%.

### 4.11. Statistical Analysis

All data were expressed as mean ± SD with the statistical method of one-way analysis of variance (ANOVA) and Student’s *t*-test using the SPSS 19.0 software. A *p* value less than 0.05 was considered as a statistically significant difference. Metabolomics data was analyzed based on the technique described in our previous study [12].

## 5. Conclusions

In summary, our studies showed that EO exerts anti-inflammatory effects by inhibiting iNOS gene expression and NO production, as well as the expression and secretion of inflammatory cytokines (IL-6, IL-18, IL-1β, and TNF-α) in LPS-treated BV2 microglial cells. LPS promotes significant metabolic changes, resulting in increasing glycolysis, whereas EO suppresses the increase of glucose consumption and lactate production in LPS-stimulated BV2 microglial cells, which was related to the decreasing activities of key enzymes in glycolysis. Therefore, these results suggested that EO also acts as an anti-inflammatory agent via attenuating the LPS-induced glycolysis capacity. Furthermore, these data suggested a molecular pathway that directly links glycolysis to the anti-inflammatory program of BV2 microglial cells, suggesting a potential role for metabolic therapies in treating inflammation.

## Figures and Tables

**Table 1:** Primer sequences.

Primer	Sequences
IL-18	5′-GCCTGTGTTCGAGGATATGACT-3′
	5′-CCTTCACAGAGAGGGTCACAG-3′
IL-1β	5′-GCCCATCCTCTGTGACTCAT-3′
	5′-AGGCCACAGGTATTTTGTCG-3′
IL-6	5′-AGTCACAGAAGGAGTGGCTAA-3′
	5′-GGCATAACGCACTAGGTTT-3′
iNOS	5′-GGAGTGACGGCAAACATGACT-3′
	5′-TCGATGCACAACTGGGTGAAC-3′
TNF-α	5′-ACAGCCAGGCTTCGTTTAGG-3′
	5′-GCCAATTTCGGACTCAGCATC-3′
GAPDH	5′-GGTGAAGGTCGGTGTGAACG -3′
	5′-CTCGCTCCTGGAAGATGGTG-3′

## Supplementary Materials

The following are available online. Figure S1: GC chromatogram of EO, Table S1: Chemical composition of EO.
