# Supplementary material for: Anti-Inflammatory Property of the Essential Oil from Cinnamomum camphora (Linn.) Presl Leaves and the Evaluation of Its Underlying Mechanism by Using Metabolomics Analysis
Source: Molecules. 2020 Oct 19;25(20):4796. doi: 10.3390/molecules25204796 (PMC7587563; doi:10.3390/molecules25204796)
Supplement: Supplementary file 1 [file molecules-25-04796-s001.pdf]

Supplementary Materials

Article

Anti-Inflammatory Property of the Essential Oil from *Cinnamomum camphora* (Linn.) Presl Leaves and the Evaluation of Its Underlying Mechanism by Using Metabolomics Analysis

Jiali Chen [1,2], Cailin Tang [1], Yang Zhou [1], Rongfei Zhang [1], Shaoxia Ye [1], Zhimin Zhao [1], Ligen Lin [2,*] and Depo Yang [1,*]

[1] School of Pharmaceutical Sciences, Sun Yat-Sen University, Guangzhou 510006, China; chenjli26@mail2.sysu.edu.cn (J.C.); tangclin@mail2.sysu.edu.cn (C.T.); zhouy596@mail2.sysu.edu.cn (Y.Z.); zhangrf7@mail2.sysu.edu.cn (R.Z.); yeshx9@mail2.sysu.edu.cn (S.Y.); zhaozhm2@mail.sysu.edu.cn (Z.Z.)

[2] State Key Laboratory of Quality Research in Chinese Medicine, Institute of Chinese Medical Sciences, University of Macau, Avenida da Universidade, Taipa, Macau 999078, China

* Correspondence: ligenl@um.edu.mo (L.L.); lssydp@mail.sysu.edu.cn (D.Y.); Tel.: +853-88228041 (L.L.); +86-020-39943043 (D.Y.)

Academic Editors: Elisa Ovidi and Antonio Tiezzi

Received: 19 September 2020; Accepted: 16 October 2020; Published: 19 October 2020

Table S1. Chemical composition of EO.

No	Compounds	RI	Percentage (%)
1	Linalool	1099	31.85%
2	Eucalyptol	1032	19.16%
3	Isoborneol	1157	9.47%
4	α-Terpineol	1190	8.47%
5	Camphor	1144	4.78%
6	Bicyclo[3.1.0]hexane, 4-methylene-1-(1-methylethyl)-	974	4.22%
7	Caryophyllene	1419	2.86%
8	Humulene	1579	1.58%
9	α-Pinene	937	1.48%
10	Naphthalene	1486	1.33%
11	(-)-Spathulenol	1576	1.22%
12	Terpinen-4-ol	1182	1.06%
13	β-Pinene	937	1.05%
14	Bicyclo[3.1.1]heptane, 6,6-dimethyl-2-methylene-, (1S)-	943	1.05%
15	5-Isopropyl-2-methylbicyclo[3.1.0]hexan-2-ol	1075	0.98%
16	Germacrene D	1432	0.95%
17	1-Hexanone, 1-phenyl-	1473	0.95%
18	Propanoic acid, ethyl ester	710	0.92%
19	γ-Muurolene	1477	0.80%
20	β-Ocimene	1038	0.48%
21	Cyclohexane	1391	0.33%
22	2-Carene	1001	0.31%
23	Camphene	952	0.29%
24	γ-Elemene	1434	0.28%
25	Caryophyllene oxide	1581	0.28%
26	Cis-Linaloloxide	1086	0.27%
27	α-Phellandrene	1005	0.18%
28	cis-3-Hexenyl iso-butyrate	1145	0.18%

29	β-Myrcene	991	0.17%
30	2,2'-Isopropylidenebis(5-methylfuran)	1458	0.15%
31	Copaene	1376	0.14%
32	(+)-4-Carene	1009	0.13%
33	Methyl Isobutyl Ketone	735	0.09%
34	4-Indolyl acetate	1554	0.08%
35	trans-Calamenene	1529	0.07%
36	γ-Terpinene	1060	0.06%
37	Benzenamine, N-(1-methylethyl)-	1127	0.06%
38	Ylangene	1372	0.06%
39	Longipinane	1398	0.06%
40	β-Eudesmol	1649	0.06%
41	Trans-Carveol	1090	0.05%
42	Isospathulenol	1549	0.05%
43	1-Propen-2-ol, acetate	609	0.04%
44	Neointermedeol	1660	0.04%
45	n-Propyl acetate	708	0.04%
46	Aromandendrene	1440	0.04%
47	9-Ethylbicyclo(3.3.1)nonan-9-ol	1292	0.04%
48	endo-Borneol	1167	0.04%
49	Others		1.75%
**Total**			100 %

RI: the retention index was calculated for all volatile constituents using a homologous series of n-alkanes C7-C40 on DB-5MS column.

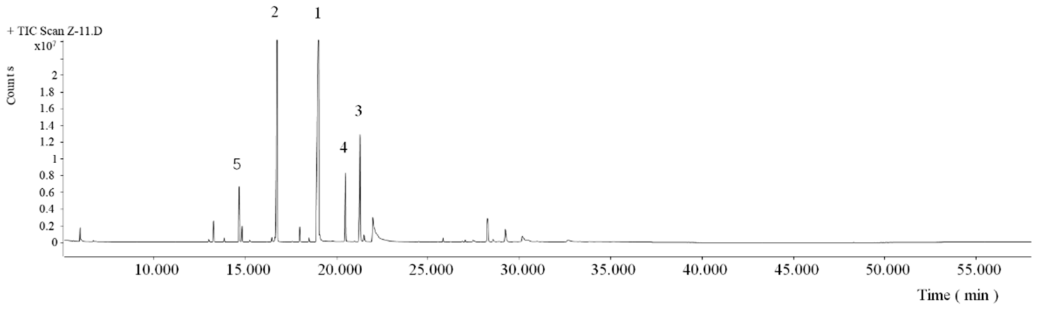

Figure S1. GC chromatogram of EO.

Linalool（1）Eucalyptol（2）Isoborneol（3）*α*-Terpineol（4）Camphor（5）

The composition of EO was analyzed by an Agilent 7890A gas chromatographic (GC) instrument coupled to an Agilent 5975 mass spectrometer (MS) (Agilent Technologies, Inc., Santa Clara, CA, USA), equipped with an DB-5MS capillary column (40.00 m × 250.00 μm × 0.25 μm film thickness). The carrier gas, helium, was injected at a constant flow rate of 1.0 mL/min. The temperature of injector was 280 °C. The sample (1 μL) was injected in the split mode (1:5). The initial oven temperature was 40 °C and was maintained at 40 °C for 5 min, then the oven temperature would be gradually raised to 280 °C at 5 °C /min and was finally maintained for 5 min. The mass spectrometer was operated at an ionization voltage of 70 eV and a mass range of 40-600 *m*/*z*.
